# Gut Microbiota of Ruminants and Monogastric Livestock: An Overview

**DOI:** 10.3390/ani15050758

**Published:** 2025-03-06

**Authors:** Giuseppe Tardiolo, Deborah La Fauci, Valentina Riggio, Matteo Daghio, Eleonora Di Salvo, Alessandro Zumbo, Anna Maria Sutera

**Affiliations:** 1 Department of Veterinary Sciences, University of Messina, Viale Giovanni Palatucci 13, 98168 Messina, Italy; gtardiolo@unime.it (G.T.); deborah.lafauci@unime.it (D.L.F.); 2 The Roslin Institute and Royal (Dick) School of Veterinary Studies, University of Edinburgh, Easter Bush Campus, Edinburgh EH25 9RG, UK; valentina.riggio@roslin.ed.ac.uk; 3 Department of Agriculture, Food, Environment and Forestry, University of Florence, Piazzale delle Cascine 18, 50144 Florence, Italy; matteo.daghio@unifi.it; 4 Department of Biomedical, Dental Sciences, Morphological and Functional Imaging, University of Messina, Via Consolare Valeria 1, 98125 Messina, Italy; eleonora.disalvo@unime.it; 5 Department of Chemical, Biological, Pharmaceutical and Environmental Sciences, University of Messina, Viale Ferdinando Stagno d’Alcontres 31, 98166 Messina, Italy; asutera@unime.it

**Keywords:** gut microbiota, ruminants, monogastric animals, livestock

## Abstract

The diversity and composition of the gut microbiota are widely recognized as fundamental factors influencing the well-being and productivity of domestic animals. Advancements in sequencing technologies have revolutionized studies in this research field, allowing for deeper insights into the composition and functionality of microbiota in livestock. Ruminants and monogastric animals exhibit distinct digestive systems and microbiota characteristics: ruminants rely on fermentation, while monogastrics use enzymatic digestion, and monogastric animals have simpler stomach structures, except for horses and rabbits, where both processes coexist. Understanding the gut microbiota’s impact and composition in both animal types is essential for optimizing production efficiency and promoting animal health. Following this perspective, the present manuscript review aims to provide a comprehensive overview of the gut microbiota in ruminants (such as cattle, sheep, and goats) and monogastric animals (including horses, pigs, rabbits, and chickens).

## Simple Summary

The gut microbiota has a crucial role in livestock by aiding gut development, digestion, and immune responses. Early-life microbial colonization can have long-term effects on the host’s microbiota and phenotype. The gut microbiota includes a diverse community of bacteria, archaea, fungi, and protozoa, while the microbiome encompasses these microorganisms along with viruses, plasmids, and microbial structural components. Advances in sequencing techniques have enhanced our understanding of livestock microbiota, with significant implications for animal health and production and environmental sustainability. Here, we explore the gut microbiota in both ruminants and monogastric animals, emphasizing the importance of their digestive tract in animal production and health.

## 1. Introduction

The gastrointestinal microbiota plays an active and fundamental role in a concert of important functional processes, including the development of the gut, the digestion and utilization of feed, and the immune response in livestock [1]. However, alterations in the early-life colonization of gut microbes in livestock often lead to lasting impacts on the formation of microbiota, subsequently influencing the phenotype of the host [2,3].

The microbiota constitutes a diverse ecological community encompassing commensal, symbiotic, and pathogenic microorganisms, which include bacteria, archaea, fungi, and protozoa [4]. In addition, recently defined as the amalgamation of microbiota and its associated habitat, the microbiome comprises viruses, plasmids, dead cells and extracellular DNA, and microbial structural components such as proteins, polysaccharides, nucleic acids, and more [5]. Nonetheless, this composition reflects the host’s long-term evolutionary adaptation to its dietary habits.

The gut microbiota is a complex ecosystem primarily composed of bacteria; however, other microbial groups, including bacteriophages and fungi, also play significant roles in gut homeostasis. Bacteriophages (phages), which are viruses that infect bacteria, are abundant in the gastrointestinal tract and contribute to shaping bacterial communities through predation and horizontal gene transfer [6]. Phage–bacteria dynamics can play a crucial factor in microbial ecology, influencing bacterial diversity, antimicrobial resistance, and metabolic functions [7]. By modulating bacterial populations, phages help maintain gut microbial balance and have been proposed as potential tools for microbiota engineering in livestock [8]. Similarly, fungi constitute a minor but functionally important component of the gut microbiota, particularly in ruminants, where they contribute to fibre digestion. Members of the *Neocallimastigomycota* phylum, such as *Piromyces* and *Neocallimastix*, are anaerobic fungi specialized in degrading recalcitrant plant material through the production of powerful hydrolytic enzymes [9,10,11]. These fungi establish synergistic relationships with fibrolytic bacteria and methanogenic archaea, enhancing the efficiency of lignocellulose breakdown in the rumen [12]. In monogastric animals, fungal species such as *Candida* and *Saccharomyces* are often commensals, although imbalances can lead to dysbiosis and health complications [13]. While the current review primarily focuses on the bacterial component of gut microbiota, acknowledging the interplay between bacteria, phages, and fungi is essential for a holistic understanding of microbial ecosystems in livestock species. Future research integrating multi-kingdom microbial interactions will provide deeper insights into microbiota functionality and its impact on animal health and production.

Microbial communities in the gastrointestinal (GI) tract are shaped by a multitude of environmental and physiological factors that vary across different gut compartments. These factors include pH, osmolarity, nutrient availability, oxygen levels, and feed retention time, all of which play crucial roles in selecting for specific microbial populations [14,15]. The pH gradient along the GI tract is one of the most significant determinants of microbial composition. The rumen, for instance, maintains a pH between 5.5 and 7.0, which favours the growth of fibre-digesting bacteria such as *Fibrobacter* and *Ruminococcus* [16]. In contrast, the acidic conditions of the monogastric stomach (pH 1.5–3.5) select for acid-resistant microorganisms, such as *Lactobacillus* and *Streptococcus* [17]. The osmotic pressure in the gut is influenced by diet composition and water absorption. High-starch diets lead to increased osmolarity in the intestines, which can shift microbial populations towards amylolytic bacteria such as *Prevotella* [18]. The residence time of digesta varies significantly between ruminants and monogastrics, affecting microbial fermentation efficiency. In ruminants, fermentation occurs over prolonged periods (30–50 h in the rumen) before passage to the intestines, allowing efficient fibre degradation [19]. In contrast, monogastrics exhibit shorter transit times, limiting the extent of microbial digestion. The gut is largely anaerobic, but microaerophilic niches exist in certain regions (e.g., the epithelium-associated microbiota in the intestines). Facultative anaerobes such as *Escherichia coli* thrive in these conditions and can outcompete strict anaerobes in response to host metabolic shifts [20]. These factors collectively contribute to niche specialization and interspecies differences in microbiota composition, highlighting the complex interplay between host physiology and microbial ecology.

In recent times, the continuous improvement of sequencing techniques has facilitated remarkable progress in microbiome studies across various domains [21], enhancing our comprehension of microbiota composition and functionality in livestock [22]. In particular, the ongoing advancement in our understanding of the livestock microbiome [23] has significant potential in enhancing animal production and health, while simultaneously mitigating environmental pollution [24,25]. This prospect becomes particularly crucial when taking into account projections indicating an almost two-fold increase in meat production and consumption over the next thirty years. Changes in nutrition patterns in low- and middle-income countries, coupled with the growth of the global population, are expected to further drive the demand for dairy products [26]. The vast majority of animal products consumed by humans come from key livestock species [27].

Distinct differences exist between ruminants, characterized by a multi-chambered stomach utilized for the fermentation-based digestion of plant materials, and monogastric animals, whose stomach is a single-compartment structure [28]. Ruminants, such as cattle, sheep and goats, possess a complex stomach with four compartments—the rumen, reticulum, omasum, and abomasum—facilitating the fermentation processes of fibrous plant materials prior to digestion [29]. In contrast, monogastric animals, including horses, pigs, rabbits, and poultry, have a single-chambered stomach where digestion occurs primarily through enzymatic action rather than microbial fermentation [30,31]. This fundamental difference impacts the composition and function of the gut microbiota between these groups, necessitating a broad consideration of the entire gastrointestinal tract when discussing gut microbiota in ruminants [32]. Therefore, it is crucial to clearly highlight these differences to prevent confusion, especially when addressing audiences with diverse scientific backgrounds [33]. For example, the benefits of ruminants being able to derive energy from low-quality food and the challenges associated with maintaining a balanced and healthy ruminal microbiota do not apply to monogastrics. Monogastric animals are known for their quicker development and shorter lifespan compared to the more intricate digestive system of ruminants [34]. Based on what is known to us today about the gastrointestinal microbiota, the present review aims to provide an overview focused on the gut microbiota of both animals with multi-chambered stomachs and monogastric animals. Additionally, existing knowledge gaps, including breed-specific differences and the current state of the art of microbiota in indigenous breeds are reported.

## 2. Gut Microbiota in Ruminants

Cattle (*Bos taurus*), sheep (*Ovis aries*), and goats (*Capra hircus*) are herbivores with a specialized stomach structure that allows for the fermentation of fibrous plant materials. These species, while sharing a common digestive system, exhibit distinct physiological traits and environmental adaptations that shape their gut microbiota. Understanding the microbial composition and functionality in the gastrointestinal tract (GIT) of these animals is critical for enhancing health and productivity in livestock systems [35].

**Figure 1:**
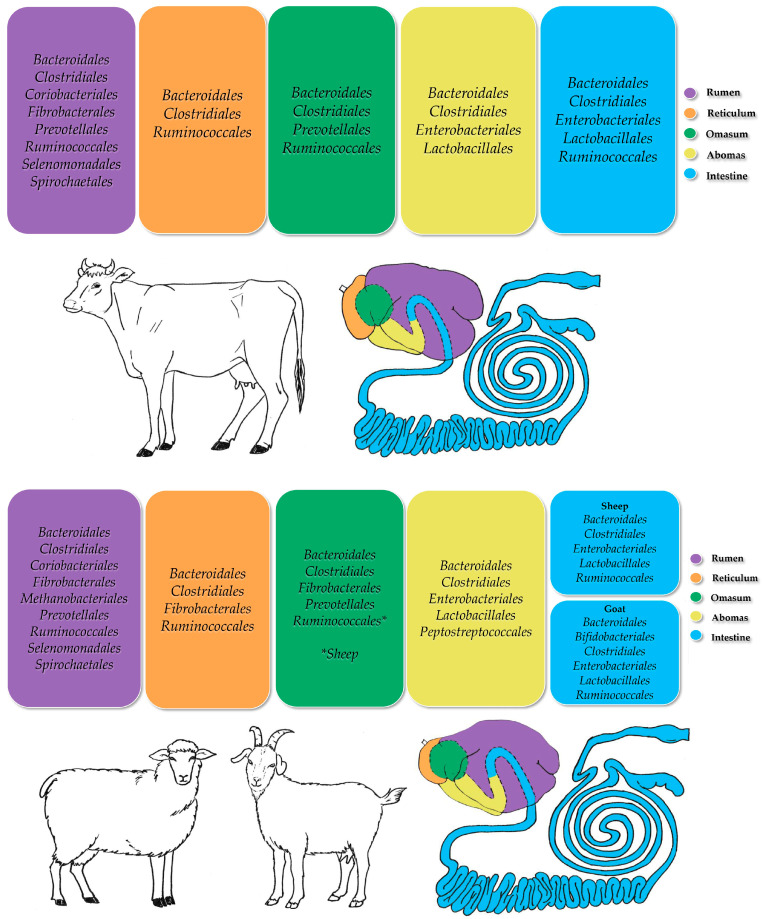
Representation of the predominant bacterial orders identified in the gastrointestinal tracts of ruminant livestock species. The term “intestine” is used here to encompass both the small and large intestines for clarity (adapted from Forcina et al. [48]).

Rumen microorganisms can be divided into three fractions according to their location: liquid-associated microbiota, solid-associated microbiota, and rumen-epithelium-associated microbiota [36]. Research on the ruminant gut microbiome has primarily focused on characterizing microbial communities through 16S rRNA gene sequencing. This has provided insights into the microbial diversity influenced by factors such as diet composition [37], location within the GIT [38], feed efficiency [39], breed-specific characteristics [40], metabolic disorders [41], temporal changes [42], individual variations [43,44], and different housing systems [45]. Notably, core microbial populations within the bovine rumen and other livestock species have been identified, despite variations across individuals, breeds, and environments [32,46]. Additionally, despite differences in taxonomic composition (Figure 1), microbial communities often exhibit functional similarities in metabolic networks [47], emphasizing the complexity of the ruminant microbiome.

### 2.1. Cattle

Cattle have been the focus of extensive research on the gut microbiome due to the direct implications it has on productivity, health, and environmental impact. Recent studies have provided a detailed understanding of the microbial communities residing in the bovine GIT, revealing not only the taxonomic diversity but also the functional capacities of these microorganisms [49]. This section delves into the current knowledge on the bovine rumen microbiome, its structure, and its role in influencing key physiological and metabolic processes. A comprehensive survey involving dairy cows revealed that the fundamental composition of the rumen microbiome not only underlies animal productivity but also influences the nature of their emissions [50]. The shotgun metagenomics approach recently provided an avenue for an in-depth exploration of the rumen microbiome in cattle, thus allowing the assembly of complete bacterial genomes, most of which belong to novel taxa, and the discovery of new enzymes [51]. Additionally, this method has facilitated the understanding of the intricate interplay between the rumen microbiome, its metabolome, and the host metabolome. This has shed new light on the nuanced mechanisms that govern production performance in dairy cows [52].

In contrast to other ruminant livestock species, the exploration of the cattle gut microbiome has been notably comprehensive, offering a detailed background of the bacterial communities residing in various locations within the GIT [53]. The most prevalent phyla in the GIT bacterial community include *Bacteroidetes* and *Firmicutes* (also referred to as *Bacillota*, [54]), collectively constituting over 90% of the entire composition [55]. *Actinobacteria*, *Proteobacteria*, *Spirochaetes*, and *Tenericutes* represent additional major taxa, albeit in comparably lower abundances [45,56]. *Bacteroidetes* and *Firmicutes* are primarily dominated by the classes *Bacteroidia* and *Clostridia*, alongside *Bacilli*. In terms of major orders, *Bacteroidales* is predominant in the former class, while *Clostridiales* prevails in the latter [51]. Prominent families within the GIT bacterial community encompass *Bacteroidaceae*, *Clostridiaceae*, *Lachnospiraceae*, *Peptostreptococcaceae*, *Rikenellaceae*, and *Ruminococcaceae* [32,45].

Among the dominant genera, prevalent not only in cattle but in adult ruminants overall [32], are *Butyrivibrio*, *Prevotella*, and *Ruminococcus* [52,57]. The genus *Clostridium* is also abundant in the cattle rumen [56], alongside *Acetitomaculum*, *Acinetobacter*, *Mogibacterium*, *Succiniclasticum*, and *Treponema* [38]. Interestingly, it has been highlighted that genera like *Fibrobacter* and *Ruminococcus* are core heritable bacteria, transferred vertically across generations due to their pivotal role in the cellulolysis process [50].

*Archaea* represent a key component of the cattle gut microbiome, particularly within the rumen, where they play a role in methanogenesis. The predominant archaeal taxa in cattle belong to the phylum *Euryarchaeota*, with genera such as *Methanobrevibacter* and *Methanosarcina* being the most abundant [58,59,60]. These methanogenic archaea utilize hydrogen and carbon dioxide to produce methane, a key byproduct of rumen fermentation that contributes to enteric methane emissions [32]. The interactions between archaea and bacterial communities in the rumen are essential for maintaining microbial homeostasis and optimizing fermentation efficiency. Archaea establish syntrophic relationships with fibrolytic bacteria, such as *Fibrobacter succinogenes* and *Ruminococcus flavefaciens*, which degrade complex plant polysaccharides into fermentable substrates [61]. These metabolic interactions facilitate hydrogen transfer, preventing its accumulation and ensuring efficient volatile fatty acid (VFA) production, which is critical for ruminant energy metabolism [62].

Given their role in methane production, *Archaea* have been a major focus of research on mitigation strategies aimed at reducing enteric methane emissions. Various approaches, including dietary modifications (e.g., supplementation with fats, tannins, or essential oils), microbial inhibitors, and the use of alternative electron acceptors, have been explored to manipulate rumen microbial composition and lower methane output [63,64]. However, further research is needed to understand the resilience and adaptability of the rumen archaeal community under different feeding regimens and environmental conditions.

Different factors can influence the rumen microbiome, and among them, the diet is the main driving factor. Due to the increasing interest in extensive farming systems, the microbiota composition of grazing cattle has been compared to that of cattle raised in intensive systems. Grazing cattle exhibit distinctive microbiota profiles compared to those raised in feedlot systems, mainly due to differences in diet composition and forage availability [40,65]. The shift from a high-forage diet in grazing systems to a high-grain diet in feedlots leads to significant alterations in microbial community structure, favouring amylolytic bacteria such as *Succinivibrio* and *Streptococcus bovis* at the expense of fibrolytic bacteria [66]. Additionally, pasture-based systems have been associated with increased microbial diversity and enhanced resilience to dietary stress, which can contribute to improved animal health and performance [32]. Environmental factors, such as soil composition and climate conditions, also play a crucial role in shaping the gut microbiota of grazing cattle, influencing microbial interactions and nutrient metabolism [67].

### 2.2. Sheep

Over the last decade, interest in studying the microbiome in sheep has grown significantly. It has been proposed that different feeding strategies might encourage a more or less diverse microbial community [68]. Moreover, alterations in microbiome composition have been noted along the GIT [69,70], and they have been linked to parasite infections [71].

The shifts in the archaeal community, rather than the eubacterial community, appear to play a significant role in feed efficiency in sheep. In a study comparing rumen bacterial and archaeal populations of sheep divergent for feed conversion ratio, McLoughlin et al. have indeed shown that feed efficiency is likely influenced by compositional changes to the archaeal community [72].

The microbiome composition in sheep and goats was compared, revealing no substantial differences between the two species. However, variations were observed based on age, with older individuals exhibiting higher microbial diversity, mirroring findings in Tibetan sheep [70]. Variations in gut bacterial compositions have been noted among different Chinese sheep breeds indigenous to the Tibetan Plateau [73], in contrast to findings from a similar study involving Italian sheep, where microbiome differences were predominantly attributed to distinct husbandry practices [74]. Feed efficiency has been correlated with higher abundance and diversity of rumen microbiomes [75], in contrast with dairy cattle, where a higher feed efficiency has been correlated to microbiomes with lower diversity [76,77]. Other investigations have focused on local breeds of significant socioeconomic importance, often revealing a notably diverse composition, such as in the case of Chinese Mongolian sheep [78]. Alternatively, similar to findings in cattle and goats [79], there is evident heterogeneity across various GIT locations, as evidenced in Qinghai semi-fine wool sheep [70].

Further studies exploring the potential association between host genetics and rumen microbiota in local sheep breeds have uncovered the modulating effect of ovine candidate genes on microbiome composition [80], as well as the interplay between this and host gene expression in maintaining homeostasis in extreme environments [81]. However, all previous investigations have relied on 16S metabarcoding, with limited applications of shotgun metagenomics to characterize gut microbial composition in sheep. Nevertheless, a noteworthy study combining both approaches with metaproteomics has been conducted to explore the relationship between microbial communities and biochemical pathways [82]. Interestingly, the efficacy of faecal microbiota transplantation (FMT) in relieving milk replacer-induced diarrhoea in lambs showed that FMT from healthy donor lambs significantly alleviated diarrhoea symptoms. Furthermore, FMT reduced serum inflammatory markers and colonic inflammation, thus encouraging the application of FMT in mitigating diarrhoea and modulating gut microbiota composition and metabolites in lambs [83].

Analysis of the GIT microbiome has unveiled its substantial resemblance in composition to that of cattle and other ruminants. *Bacteroidetes* and *Firmicutes*, which play key roles in fibre digestion and VFA production [84], collectively comprise over 80 to 90 percent of the gut microbial community [68,72], followed by *Actinobacteria*, *Proteobacteria*, *Spirochaetes*, and *Verrucomicrobia* [73,85,86]. In addition, *Bacteroidia* and *Clostridia* are the predominant classes [74]. Furthermore, *Bacteroidales* and *Clostridiales* are among the most abundant orders, while, akin to observations in cattle, *Eubacteriales* and *Lactobacillales* are notable within *Firmicutes*. At the family level, *Ruminococcaceae* and *Lachnospiraceae* are prominent [73], alongside *Prevotellaceae*, *Rikenellaceae*, and *Succinivibrionaceae* [72,75,81]. Regarding the most prevalent genera, *Prevotella* stands out [81], followed by *Acinetobacter* [80], *Campylobacter* [74], *Treponema*, *Ruminococcus*, *Oscillospira*, *Desulfovibrio*, *Bacteroides* [78], *Fibrobacter*, and *Succinivibrio* [75].

The gut microbiota of sheep exhibits a complex and dynamic composition influenced by various factors such as diet, breed, and environmental conditions [87,88]. However, interindividual variability in microbial diversity has been observed, particularly in response to grazing patterns and feeding regimes. Sheep raised in intensive systems often display lower microbial diversity compared to those in extensive grazing environments, where dietary diversity promotes a richer microbial ecosystem [89]. Additionally, seasonal variations significantly impact microbiota composition, with distinct shifts observed in bacterial taxa between summer and winter grazing periods [90].

### 2.3. Goats

Despite the economic significance of goat meat and dairy products, molecular studies aimed at delineating gut microbiome composition in this particular livestock species are relatively limited. This could be a consequence of the fact that microbiome research is mostly conducted in highly developed countries, where goats have little economic importance. Nonetheless, there are noteworthy exceptions, such as a study exploring the impact of dietary nitrate supplementation on microbial composition and ruminal fermentation. This investigation utilized a combined metabarcoding approach employing 16S rRNA gene and 18S rRNA gene amplicon libraries to analyse bacteria and protists, along with fungi, respectively [91]. Other studies have highlighted the influence of fatty acid supplementation [92] and a diet rich in grains [93] on shaping the bacterial and fungal diversity of the rumen microbiome, employing 16S rRNA gene and ITS metabarcoding techniques, respectively. Another study revealed the significant role played by specific fungal and bacterial consortia in facilitating lignocellulose breakdown through the production and interaction of specific metabolites [94]. Moreover, diets rich in condensed tannin-containing pine bark have been correlated to diversity in methanogenic archaea based on 16S rRNA gene analysis [95].

Further research has demonstrated that, similar to sheep and cattle, the microbial community in goats varies across different segments of the GIT [30], and fluctuations in microbial diversity with age were observed in young individuals [96], consequently enhancing their productivity [30,97]. Interestingly, the introduction of rumen fluids during early developmental stages has been observed to enhance the maturation of the rumen microbiome and expedite the weaning process [98], even if contrasting results are reported when other animals (i.e., calves) are considered [99]. Conversely, the presence of apicomplexan parasites in young goat offspring has been linked to a reduction in butyrate-producing bacteria abundance, resulting in heightened mucosal inflammation and tissue repair [100]. In contrast, antibiotic-induced dysbiosis of the gut microbiota has been suggested to exacerbate disease conditions by promoting inflammatory immune responses [101].

Variation in microbial composition has been observed when comparing adult goats from different breeds [86], although diet and environmental factors seem to have a more significant impact on microbial diversity than genotype [102]. Furthermore, the presence of specific bacterial hosts has been associated with the digestibility of dietary phosphorus [103]. However, the investigation of gut microbiome components beyond bacteria is relatively limited in goats, with one notable exception being a study utilizing 16S rRNA gene and 18S rRNA gene amplicon libraries to explore bacterial and ciliate protozoal diversity, respectively. This study examined the effects of antibacterial peptides on rumen fermentation function [104]. On the other hand, the use of shotgun sequencing techniques to characterize the gut microbiome in goats remains largely unexplored [79].

Regarding the bacterial composition, *Bacteroidetes* and *Firmicutes* emerge as the prominent phyla, collectively constituting over 80% of the GIT bacterial community [96,103], followed by *Proteobacteria*, *Verrucomicrobia*, *Fibrobacteres*, *Spirochaetes*, and *Tenericutes* [30,92,93]. Dominant orders such as *Bacteroidales* and *Clostridiales* predominate over others [70], as also observed in cattle and sheep. *Prevotellaceae*, *Veillonellaceae*, and, to a lesser extent, *Lachnospiraceae*, *Rikenellaceae*, and *Ruminococcaceae* emerge as the dominant families [92,103]. *Prevotella*, along with *Bacteroides*, *Butyrivibrio*, *Clostridium*, *Oscillospira*, *Ruminococcus*, *Succiniclasticum*, and *Succinivibrio*, are among the prevalent genera [30,78,86,92,93,96,105].

Goats share a core microbiota with other ruminants but exhibit notable distinctions due to their feeding behaviour and dietary adaptability. As intermediate feeders, goats consume a more diverse range of forage compared to cattle and sheep, which is reflected in their gut microbiota composition [92,93]. Studies have shown that *Ruminococcaceae* and *Prevotellaceae* are among the dominant bacterial families in goat microbiota, contributing to efficient fibre degradation and metabolic flexibility [106]. The ability of goats to digest tannin-rich plants, which are often avoided by other ruminants, is linked to the presence of tannin-resistant microbial populations, such as certain strains of *Butyrivibrio* and *Pseudobutyrivibrio* [106]. Furthermore, microbial diversity in goats varies between pastoral and stall-feeding systems, with higher alpha diversity observed in animals raised under free-grazing conditions [107].

## 3. Gut Microbiota in *Monogastric* Species

**Figure 2:**
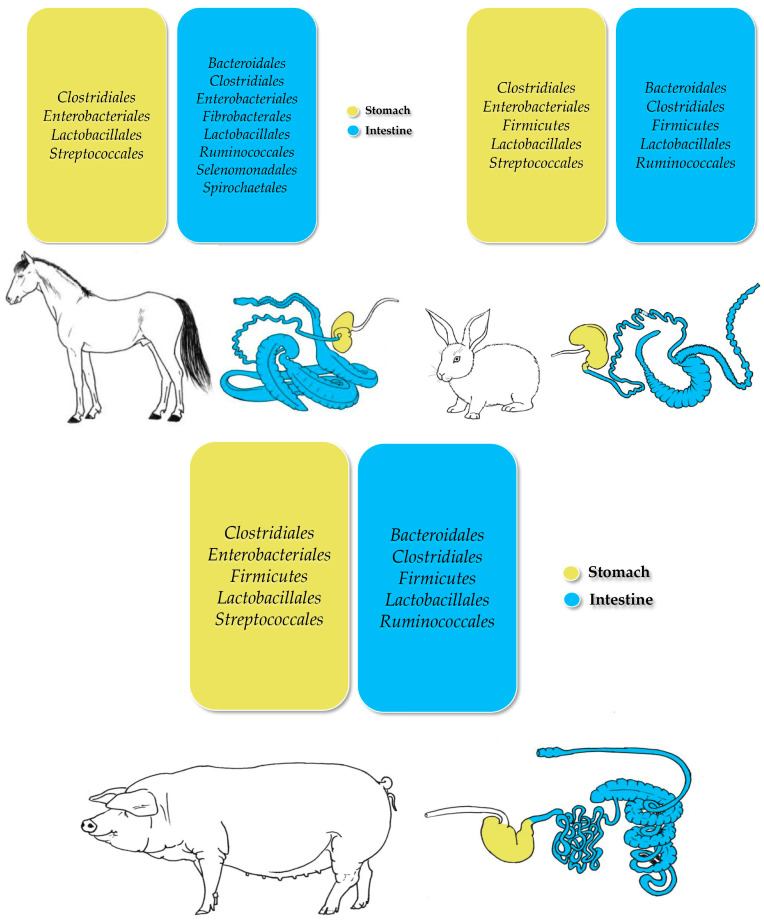
Representation of the most prevalent bacterial taxa detected in the gastrointestinal tracts of monogastric livestock species (adapted from Forcina et al. [48]).

Horses (*Equus ferus caballus*), pigs (*Sus scrofa domesticus*), rabbits (*Oryctolagus cuniculus*), and various poultry species possess a simpler digestive system characterized by a single-chambered stomach (Figure 2). This anatomical structure influences their feeding strategies, nutrient absorption, and gut microbiota composition. Unlike ruminants, monogastric animals primarily rely on enzymatic digestion to break down carbohydrates, proteins, and fats, which can affect the diversity and function of their gut microbial communities [108].

Research into the gut microbiome of monogastrics has accelerated, revealing the complex interplay between diet, microbiota, and host health. Factors such as diet composition [109], age [23,110], and environmental conditions [111] significantly shape the microbial populations in these species. Similarly, the gut microbiome plays a crucial role in fibre digestion and overall metabolic health in horses [112], while rabbits rely on specific microbial populations to efficiently ferment plant materials in their caecum [52]. In poultry, the gut microbiota is essential for nutrient utilization and immunity, influencing growth rates and disease susceptibility [113]. Understanding the microbial ecosystems within these monogastric species is crucial for improving animal welfare, optimizing production systems, and developing strategies to mitigate environmental impacts associated with livestock farming.

### 3.1. Horses

Similar to other mammals, the gut microbiome of horses creates a sophisticated and mutually beneficial environment hosting a diverse microbiota comprising bacteria, fungi, protozoa, and archaea [114]. Besides helping with food digestion, it performs metabolic functions that protect against pathogens and boost the immune system.

Changes in the microbiome composition, termed dysbiosis [115], have been linked to various factors such as diets rich in concentrates, low-quality forage, confinement, stress, fasting, and age. Interestingly, several studies have linked changes in the gut microbiome of horses to colic, as shifts in the abundance of specific bacterial groups crucial for maintaining gastrointestinal health have been observed [116]. These microbial communities actively engage with the host to uphold gut health. On one hand, the host gut provides optimal environmental conditions for microbial flourishing, including nutrients, temperature, humidity, and pH [117]. Simultaneously, the gut microbiota ferments complex carbohydrates into short-chain fatty acids, thus providing the host with indispensable nutrients and energy [118]. Furthermore, certain beneficial microbes within the gut have the capacity to specifically adhere to the mucosal epithelium, bolstering the gut’s immune defence barrier and thwarting the intrusion of pathogenic microorganisms [119].

The horse GIT exhibits an increase in richness and evenness towards its distal portions, reflecting the intricate nature of this environment [120]. It is plausible that a core microbiota consisting of microbial taxa shared among most, if not all, horses may be present across various segments of the GIT, with notable distinctions observed between the foregut and hindgut regions [117]. Several studies indicate that the bacterial communities in faeces do not significantly differ from those in the colon [120] or even the caecum [121] but do differ from those in the upper tract. Therefore, the study of faeces can provide intriguing, albeit incomplete, non-invasive markers reflecting the conditions of the hindgut (particularly the colon) rather than the foregut.

Studies conducted in healthy equines have demonstrated that *Firmicutes* can constitute as much as 45–68% of the gut microbiota, while *Bacteroidetes* may range from 14 to 37% [122]. However, contrasting results were reported in another study, which found higher levels of *Bacteroidetes* compared to *Firmicutes* in both healthy young horses and older horses, including those with colitis [123]. *Proteobacteria*, a phylum of Gram-negative bacteria, ranks as the second most prevalent group in the equine gastrointestinal tract, particularly abundant in the upper regions and peaking in the ileum at around 33%. Elevated levels of *Proteobacteria* have been linked to inflammatory intestinal conditions and dysbiosis, such as equine colic [124]. Alternative approaches by using Targeted Enrichment Hybrid Capture (TEHC) have also been explored, indicating that such technology offers superior microbial community characterization compared to conventional 16S rRNA gene amplicon sequencing methods [125]. TEHC consistently identifies more genera and species in equine faecal samples, potentially capturing bacterial species missed by 16S rRNA gene amplicon sequencing. While TEHC generates more operational taxonomic units (OTUs), this may be due to increased coverage of the 16S rRNA gene sequence rather than detecting more bacterial species. TEHC also reveals a richer microbial diversity at the phylum level, detecting additional phyla such as *Tenericutes* and *Euryarchaeota*. Overall, TEHC provides a more accurate and comprehensive characterization of the equine faecal microbiome, suggesting its potential as a preferred sequencing strategy for microbiome studies [125].

Cooke et al. [126] investigated the effects of probiotics and prebiotics on the composition of the equine faecal and seminal microbiomes and sperm quality, highlighting potential benefits of probiotics and prebiotics on equine gut health, semen quality, and increasing in microbial diversity. The faecal microbiota composition was influenced by dietary factors, including the type of forage. Specific dietary interventions, such as supplementation with probiotics and prebiotics, likely influenced the equine faecal microbiota composition. *Firmicutes*, *Bacteroidetes*, and *Verrucomicrobia* were the most abundant in the faeces of all miniature pony stallions for all treatments and time points.

### 3.2. Pigs

Microbiome research within the swine farming sector has been driven by the necessity to mitigate animal stress, which could otherwise result in financial losses for farmers [127]. The period of weaning is particularly crucial as it involves a significant dietary transition for piglets.

Investigations into the gut microbiome of pigs have greatly benefited from the development of a comprehensive gene database through extensive metagenome sequencing of faecal samples [33]. These studies have reaffirmed that, similar to other livestock species, the interaction between diet and gut function throughout various growth stages influences animal health and productivity [128,129], including aspects such as fat accumulation [109]. The microbial diversity in pigs exhibits variability based on several factors, including diet [94], gastrointestinal tract location [130,131], behaviour [132], parasite infections [133], breed [129,134], and sex [135]. Additionally, research indicates a positive correlation between microbial diversity and piglet weight and age [92,136]. Furthermore, investigations utilizing both 16S rRNA gene metabarcoding and shotgun metagenomic sequencing have demonstrated significant and predictable variations in the composition of the pig gut microbiome throughout its lifespan [137]. Particularly following weaning, there is an observed increase in microbial diversity along with higher levels of genes associated with oxidative stress and heat shock compared to pre-weaning stages [138,139].

Some studies have shown that a combination of culturomics and shotgun metagenomics can provide a more comprehensive understanding of gut microbiota and antimicrobial resistance in pigs [140,141,142]. Additionally, the utilization of 18S rRNA gene metabarcoding on faecal samples has facilitated the compilation of a comprehensive inventory of intestinal protist parasites [143]. Within the pig gut, the integration of 18S rRNA gene and ITS amplicon libraries has been utilized to characterize the community of microbial eukaryotes, revealing associations between certain members of this community and the host’s body weight [144]. Furthermore, the combination of 16S rRNA gene amplicon data and metagenomics has yielded unparalleled understanding of the functional and taxonomic diversity present within the pig gut microbiome [143].

Despite the notable differences in the structure of the GIT between ruminants and monogastric animals like pigs, the gut microbiome of the latter is primarily characterized by the phyla *Bacteroidetes* and *Firmicutes* [136], followed by *Proteobacteria* [86,132]. The most abundant classes include *Bacteroidia*, *Clostridia*, and *Bacilli* [132,144], whereas the dominant orders are *Bacteroidales* and *Clostridiales*. The most prevalent families comprise *Bacteroidaceae*, *Enterobacteriaceae*, *Lachnospiraceae*, *Lactobacillaceae*, *Prevotellaceae*, and *Ruminococcaceae* [92]. Among adult pigs, commonly observed genera in the GIT include *Alloprevotella*, *Bacteroides*, *Escherichia*, *Lactobacillus*, and *Prevotella* [130,142,145], along with *Clostridium*, *Desulfovibrio*, *Enterococcus*, *Fusobacterium*, and *Streptococcus* [143,146].

### 3.3. Rabbits

In rabbits, the intestinal microbiota consists of various microbial species, predominantly bacteria, numbering in the billions per gram and spanning over a thousand different species [147].

Rabbits excrete hard faeces (containing poorly digestible particles) and soft faeces (containing fine particles from caecum fermentation; caecotropes), which provide additional nutrients when ingested by the animal [148]. Caecotroph animals like rabbits heavily rely on the caecum for fermentation. Recent research has expanded the understanding of rabbit gut microbiota, particularly in terms of seasonal variations and health impacts [149]. Several studies have highlighted that the gut microbiota composition can be influenced by seasonal changes, affecting the productivity and health status of meat rabbits [150]. For instance, rabbits reared in semiconfined conditions exhibit significant differences in gut microbial communities between summer and winter, with dominant phyla such as *Firmicutes* and *Verrucomicrobiota* showing seasonal abundance variations. These shifts in microbiota are associated with changes in the animal’s physiology and immune responses, illustrating the complex interaction between environmental factors and gut microbiota [150].

The gut microbiota has also been linked to broader health implications beyond digestion. A recent study investigating the impact of gut microbiota on intervertebral disc degeneration in rabbits revealed that specific bacterial taxa were enriched in diseased animals compared to healthy controls. This suggests a potential role of gut microbiota in modulating systemic health conditions [151]. Understanding these microbial populations’ roles and how they interact with various factors such as nutrition, genetics, and diseases is crucial for improving rabbit health and productivity. Strategies aimed at fostering a beneficial intestinal bacterial community could enhance nutrient digestibility, energy conservation, and immune system robustness, thereby reducing disease incidence [147,152].

A comparison of faecal types of rabbits showed that hard faeces and soft faeces have a distinct microbial community. Members of *Akkermansia*, *Blautia*, and *Oscillospira* were overrepresented in the soft faecal microbiota [148]. Prevention of caecotrophy in rabbits decreased the feed utilization and growth performance and lowered serum total cholesterol and total triglycerides. The content of short-chain fatty acids in the caecum decreased if rabbits did not ingest caecotropes. Furthermore, the microbiota differed when caecotrophy was prevented, resulting in a lower abundance of *Oscillospira* and *Ruminococcus* [153].

Future research should continue to explore the physiological and metabolic pathways influenced by gut microbiota, providing deeper insights into optimizing rabbit husbandry practices for better health outcomes.

### 3.4. Chickens

In the poultry domain, shotgun metagenomics is an emerging technique with comparative investigations to evaluate the impact of dietary supplementation on enhancing the health status and productivity of broilers by promoting the diversity of their caecum microbiome [154,155]. Dietary supplementation has been explored both in traditional forms and through in ovo methods [97]. Moreover, shotgun metagenomics has facilitated the identification and characterization of new bacterial, archaeal, and bacteriophage taxa within the chicken gut microbiome, elucidating their functional roles [156]. Nonetheless, 16S rRNA gene sequencing remains a widely utilized method for comparing microbiome compositions between healthy and diseased individuals, affected by viral or bacterial infections, diverse dietary treatments, various indigenous breeds, gastrointestinal locations, rearing systems, and lifetimes [157].

Special attention has been given to enhancing growth performance through the transfer of caecal or faecal material between individuals of different age groups [97,158]. On the other hand, less emphasis has been placed on the non-bacterial constituents of the gut microbiome, with a predominant focus on potential pathogens like *Cryptosporidium* [159].

**Figure 3:**
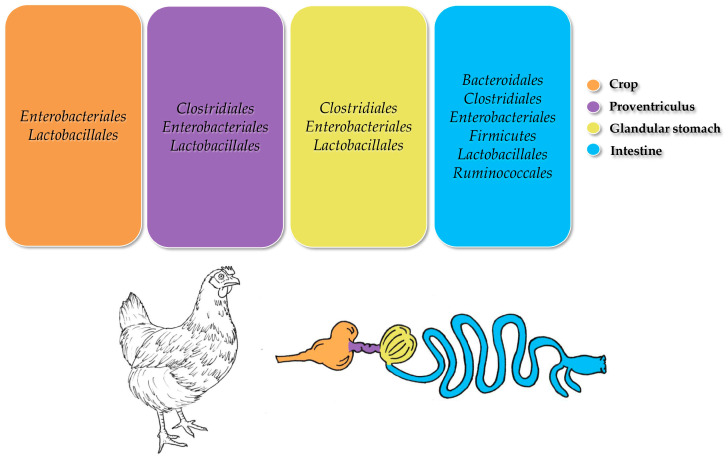
Predominant bacterial taxa observed in the gastrointestinal tracts of chickens (adapted from Forcina et al. [48]).

In the GIT tract of chickens, similar to other livestock species, the prevailing microbial phyla are typically *Bacteroidetes* and *Firmicutes*, although there are instances where *Proteobacteria* outnumber them [160]. Additionally, *Bacilli*, *Clostridia*, and *Gammaproteobacteria* are the primary classes observed [154]. At the order level, *Bacillales*, *Enterobacteriales*, *Lactobacillales*, and *Campylobacterales* are among the most frequently encountered groups [161]. Moreover, prevalent families include *Enterobacteriaceae* and *Lactobacillaceae*. Regarding dominant genera, *Alistipes*, *Bacteroides*, *Clostridium*, *Helicobacter*, *Lactobacillus*, and *Ruminococcus* are commonly identified, alongside *Flavobacterium* and notable genera such as *Campylobacter* and *Veillonella* as well [161]. Figure 3 shows the most abundant bacterial orders identified within the GIT of chickens.

## 4. Challenges in Sampling Gut Microbiota

The study of gut microbiota presents unique challenges, primarily due to anatomical constraints and sampling accessibility. Unlike rodent models or small laboratory animals, where controlled sampling is relatively straightforward, collecting biological material from ruminants and monogastric livestock often requires specialized techniques.

Faecal microbiota analysis is the most common non-invasive method for studying gut microbiota in livestock. It provides insights into luminal microbial communities, but it has limitations, as it may not fully represent mucosa-associated bacteria, which play a crucial role in host–microbe interactions [162]. Additionally, faecal samples may be influenced by transit time, diet, and environmental exposure, introducing potential biases in microbial composition assessment [49,163]. For ruminants, rumen cannulation (fistulation) is considered the gold standard for collecting ruminal contents, allowing repeated sampling from the rumen environment [164]. However, this method is invasive, requiring surgical intervention, and is mainly restricted to research settings. Alternatively, oral stomach tubing (oesophageal probe) provides a less invasive alternative but tends to underrepresent particle-associated microbes, which are crucial for fibre digestion [165,166,167]. Another approach involves the collection of gut contents and mucosal scrapings post mortem, which allows comprehensive analysis of gut segments (e.g., the rumen, caecum, and colon) and their microbiota. However, post mortem sampling is subject to rapid microbial shifts due to tissue degradation and exposure to oxygen, which can significantly alter community composition [168,169,170]. Moreover, this method does not allow for longitudinal studies, limiting its applicability in dynamic microbiota research. Endoscopic or biopsy-based sampling provides direct access to gut tissues for investigating mucosa-associated microbiota, which is crucial for understanding microbial colonization and immune interactions [162]. However, these procedures are technically challenging in large animals and often require anaesthesia or surgical intervention, limiting their practical application in large-scale studies [171].

Each sampling technique presents strengths and limitations, and the choice of method depends on the research objective, animal welfare considerations, and logistical feasibility. Future advances in non-invasive microbiota sampling (e.g., rectal swabs and metabolomic profiling) may help overcome these limitations, improving our ability to study gut microbial communities in large animals with minimal disturbance to host physiology [77]. Among the non-invasive sampling methods, recent studies have explored the use of buccal swab sampling. Kittelmann et al. [172] have shown that buccal swabs can provide an effective, non-invasive means of assessing bacterial, archaeal, and eukaryotic microbial community structures in the rumen. However, Tapio et al. [173] found that regurgitated bolus samples showed greater similarity to rumen contents than buccal swabs, possibly due to the presence of a distinct gingival microbiota. Young et al. assessed the suitability of buccal swabs as a proxy for rumen microbiota, applying time-course sampling and machine learning, showing that buccal swabs can capture key microbial taxa, though their accuracy depends on the sampling conditions and time of collection [174]. More recently, Miura et al. [175] leveraged MinION amplicon sequencing technology to improve the resolution and throughput of microbiota profiling from buccal swabs, demonstrating that this method enhances the characterization of the rumen microbiome while maintaining the advantages of a non-invasive sampling technique. These studies collectively suggest that buccal swabs represent a viable alternative to traditional rumen sampling methods, offering a practical, non-invasive approach for microbiome analysis. However, further research is needed to optimize protocols, account for site-specific microbial variability, and ensure the reliability of buccal swabs as a proxy for direct rumen content sampling.

## 5. Gut Microbiota in Nutrient Metabolism and Health

Ruminants have the capacity to make use of various residues from agriculture and the food industry as dietary resources, facilitated by the rumen microbial community [176]. With the continuous advancement of animal farming, various concerns have surfaced, encompassing matters like inefficient feed conversion, nitrogen utilization, product quality, and elevated methane discharges. These challenges are particularly conspicuous in ruminants compared to monogastric animals, owing to their distinctive digestive physiology, necessitating a deeper comprehension to tackle the aforementioned issues [177,178].

The rumen is often compared to a black box because of its complex microbial community, and the ruminal microbiota is recognized as a distinct organ made up of countless microbes, with a gene pool much larger than that of the host’s cells [179]. These microbial genes influence the host’s nutrient absorption and health through specialized metabolic pathways. Thus, the ruminal microbiota plays a crucial role in the host’s digestion and metabolic processes. Several studies have indicated that feed efficiency, nitrogen utilization, and methane emissions in ruminants are influenced by various groups of ruminal microorganisms [69,180]. Additionally, the health of the host is impacted by the rumen microbiota [38].

With advancements in metagenomics, there will likely be increased focus on the ruminal microbiota, uncovering its unique role in future studies, which could lead to the development of more effective methods to enhance production performance [53]. Rumen epithelial cells play a vital role in nutrient absorption, including VFA and vitamins. Furthermore, diverse microbial populations can colonize the rumen epithelium and can directly interact with host [36]. However, our comprehension of host–microbe interactions remains limited.

Another relevant aspect concerns the discussion surrounding the impact of gut microbial taxa on the health, welfare, behaviour, and performance of livestock under various production conditions [27,181,182]. Antibiotics are currently utilized to manage diarrhoea and enhance animal growth, but this practice contributes to bacterial resistance and antibiotic residues in meat [183]. The development of new technologies to address newborn animal diarrhoea is crucial for both livestock production and public health. Early gut microbiota activity plays a key role in optimizing immune function and growth, making microbiota colonization essential for healthy development [183]. Early interventions like oral probiotics [184], faecal microbiota transplantation [83,123], and rumen microbiota transplantation [183] can effectively alleviate common disturbances such as diarrhoea. The goal is to establish suitable antibiotic alternatives to enhance livestock health sustainably and responsibly, while tackling public health concerns associated with antibiotic use in animals [183]. It is noteworthy to delve into why studies in this field are currently in high demand and their significance in both animal farming and broader society. These studies contribute significantly to the advancement of non-antibiotic microbial therapies, such as probiotics [185], and play a crucial role in identifying biomarkers of feed efficiency. This information enables the implementation of strategies aimed at improving livestock production performance [186] and growth [98,187]. Furthermore, profiling the gut microbiota is essential for monitoring livestock health status and implementing treatments to enhance it [23], as well as for preventing the onset or worsening of pathological conditions [38]. These studies also shed light on the unique adaptations of local breeds to challenging environments [81]. In terms of maintaining host health, it is noteworthy to highlight that the gut microbiota can also be influenced by various substances, including contaminants such as bisphenols, phthalates, and mycotoxins [188]. While these contaminants can disrupt microbial communities, their specific effects and metabolic pathways are not fully understood, emphasizing the need for further study to understand their impact on microbial communities and their potential biotransformation by microbiota [188].

The enzymatic hydrolysis of complex polysaccharides is a key function of the gut microbiota, particularly in ruminants, where microbial communities work synergistically to break down fibrous plant material into metabolizable compounds. This process is mediated by Carbohydrate-Active enZymes (CAZymes), which include glycoside hydrolases (GHs), polysaccharide lyases (PLs), and carbohydrate esterases (CEs), each playing a distinct role in fibre degradation [189,190]. Among these, GHs catalyse the hydrolysis of glycosidic bonds in cellulose, hemicellulose, and pectin, enabling the microbial community to utilize plant-derived carbohydrates efficiently [191]. Members of the *Ruminococcaceae* and *Lachnospiraceae* families are known to produce multiple GHs, contributing significantly to the breakdown of structural polysaccharides [192]. Polysaccharide lyases (PLs), on the other hand, target uronic-acid-containing polysaccharides, such as pectin, and play a major role in the degradation of plant cell wall components [193]. Microbial enzymatic activity in the gut is highly dependent on diet composition, with high-forage diets promoting a greater abundance of fibrolytic bacteria and enzyme diversity [32]. Additionally, *Prevotella* species, which are prevalent in both ruminants and monogastric animals, are efficient producers of enzymes that degrade hemicellulose and pectin [194,195,196]. Advancements in metagenomic and metatranscriptomic analyses have provided deeper insights into the enzymatic potential of gut microbiota, revealing a vast repertoire of novel CAZymes with potential applications in animal nutrition and biotechnology [197]. Understanding the role of microbial hydrolases in fibre digestion is essential for optimizing feed formulations, improving nutrient utilization efficiency, and reducing environmental impacts associated with livestock production.

## 6. Gut Microbiota and Reproductive Endocrine Axis

In recent years, research has focused on studying the potential interactions between the reproductive hormonal axis and the gut microbiota. In this context, some authors explored the relationship between cyclic changes in steroid sex hormones and the gut microbiota in healthy horses during the oestrous cycle [198]. Regular fluctuations in sex hormone levels were observed during the oestrous cycle, with estradiol-17β (E2) concentrations increasing during the follicular phase (FP) and progesterone (P4) concentrations increasing during the luteal phase (LP). Analysis of faecal microbiota composition revealed stability in overall composition throughout the oestrous cycle. However, the genus *Rhodococcus*, a specific low-abundance pathogenic bacterium, increased its population during the LP compared to the FP, exhibiting changes in correspondence with sexual hormonal fluctuations [198]. It has been found that mice undergo significant shifts in their gut bacteria composition after gonad removal [199,200], and when hormone therapy was administered afterwards, it notably impacted the composition of their microbiota [200,201].

This phenomenon has also been observed in other animals like sows, where changes in sex hormone levels led to a modification in their gut microbiota [202]. In the context of functional interaction between gut microbiota and sex steroids, it is important to highlight that these molecules may also be directly synthesized in the gut [199]. Indeed, the adult male rat colon expresses steroid enzymes involved in steroidogenesis synthesis, such as P4, testosterone (T), and E2 [203]. Interestingly, the receptor for luteinizing hormone (LH) (one of the gonadotropins responsible for steroidogenesis) is not only expressed in the gonads but also in the gastrointestinal tract of humans and rats [204]. This means that the steroid gut production is partially under the control of the central reproductive axis. It has been demonstrated that levels of pregnenolone (PREG), P4, and isoallopregnanolone (ISOALLO) were higher in the female rat colon, whereas the level of T was higher in males [199].

This sexual dimorphism of gut steroidogenesis is also reported after gonadectomy, suggesting a partial autonomy of the intestinal tract in the local synthesis of sex hormones, which could be independent, within certain limits, of plasma concentration and endocrine central control. Sex and gonadectomy appear to influence microbiota composition, highlighting the complex interplay between central and local regulatory mechanisms in the synthesis of intestinal sex hormones. Some taxa and metabolic pathways were associated with gut steroids, such as positive associations between *Blautia* and T, dihydroprogesterone (DHP), and allopregnanolone (ALLO), whereas negative associations were noted between *Roseburia* and T, ALLO, PREG, ISOALLO, DHP, and P4 [199]. In humans, research indicates that the menstrual cycle, especially fluctuating sex hormones, might impact the gut microbiota composition [205]. Some authors, through taxonomic analysis, detected *Akkermansia* and *Lactococcus* in higher abundances in the LP compared to the FP, concluding that the oestrogen concentration has a complex impact on *Akkermansia* growth [206]. Wu et al. showed that reproductive hormones, in particular E2, were correlated with diversity indices and biomarkers of the shifting gut microbiota [207]. The composition and function of the intestinal microbiota were shifted during oestrus synchronization in grazing Simmental cows, and these shifts were mediated by reproductive hormones [207]. These results are in agreement with other research showing that sex hormones directly modulate the microbial metabolism through steroid receptors, including estrogen receptor beta (ERβ), underlining the importance of oestrogen-mediated intestinal microbiota shifts [208,209]. This direct effect of sex hormones on the bacterial metabolism is a regulatory mechanism that significantly affects intestinal microbial diversity [210]. Their influence also extends to controlling gene expression, protein synthesis, and multiple biological processes within the gut microbiota [211].

Although several studies have explored the putative impact of sex hormones on the composition of the gut microbiota, there is circumstantial evidence suggesting that the microbiota, in turn, may also influence the hormonal reproductive axis. These findings highlight a complex symbiotic relationship between the microbiota and its host. Indeed, sex hormones and the gut microbiota influence each other in a bidirectional manner. Within this reciprocal cross-talk, the microbial community plays an active role in modulating sex hormone levels, by metabolizing and degrading them and their related compounds, and ultimately affecting physiological responses in its host [212].

The commensal microbial community can influence sex hormone levels by means of its enzymes. As such, the term “strobolome” was introduced to describe the collection of genes in the gut microbiota that are able to convert inactive glucuronides into active oestrogens, primarily through the action of β-glucuronidases, which deconjugate oestrogens into their active forms. These active oestrogens enter the bloodstream and bind to estrogen receptors alpha (ERα) and ERβ, exerting a physiological effect [213]. In addition, the gut microbiota regulates the secondary metabolism of bile acids and inhibits their synthesis in the liver, thereby influencing sex hormone levels. This process occurs through the regulation of fibroblast growth factor 15 (FGF15) expression in the ileum that represses cholesterol 7α-hydroxylase (CYP7A1) synthesis in the hepatocytes, mechanisms that depend on the nuclear receptor for bile acids, the farnesoid X receptor (FXR). Because bile acids are involved in testosterone synthesis, the microbiota may indirectly influence testosterone levels by altering bile acid profiles [213]. A study carried out by Liu et al. in sows after weaning [214] revealed that a normal return to oestrus was characterized by increased abundances of *L. reuteri* and *P. copri* and decreased abundances of *B. fragilis*, *S. suis*, and *B. pseudolongum*. This study highlighted the involvement of *Limosilactobacillus reuteri* and *Prevotella* spp. in sow oestrus return, participating in the degradation of PREG, P4, and T, thereby promoting oestrogen biosynthesis. These authors suggest that gut microbiota dysbiosis may disturb the level of sow steroid hormones, sex-hormone-related compounds, and metabolites related to sow energy and nutrient supply, ultimately leading to the failure of oestrus return in sows [214]. This implies that steering the gut microbiota could be a promising strategy for influencing the sow oestrus return after weaning.

It has been shown that dietary probiotics supplementation in chickens increased the serum hormone levels of E2 and follicle stimulating hormone (FSH), egg quality, and ovarian development of laying hens [215]. However, in most cases, the meaning of the relationship between sex hormones and the gut microbiota remains unclear, as the observed associations may be purely correlative. Further research is needed to elucidate the real causal and bidirectional interactions between sex hormones and the gut microbiota, as well as the potential application of these interactions in the field of reproductive medicine.

## 7. Microbiota Manipulation: From Microbiome Characterization to Livestock Applications

The increasing availability of microbiome characterization tools, such as metagenomics, metatranscriptomics, and metabolomics, has deepened our understanding of gut microbial functions. However, translating microbiome insights into practical applications for livestock production remains challenging due to causality uncertainties and interindividual variability [216].

Several microbiota-based strategies have been explored to enhance feed efficiency, disease resistance, and overall productivity, including probiotic and prebiotic supplementation, dietary modulation, microbiota transplantation, and phage therapy and precision microbiome engineering. While probiotics (e.g., *Lactobacillus* and *Bifidobacterium*) and prebiotics (e.g., inulin and oligosaccharides) have been widely studied, their efficacy varies across species and diets. Some studies report improved growth rates and immune function, while others show negligible effects due to host-specific microbiota interactions [217].

Adjusting dietary fibre, protein, and fat content can shift microbial populations towards beneficial fermentation profiles. For example, high-fibre diets increase short-chain fatty acid (SCFA) production, improving gut health and metabolic efficiency [218]. Faecal microbiota transplantation (FMT) has been proposed as a method to restore gut microbial balance in livestock affected by dysbiosis. However, standardization challenges and donor–recipient compatibility issues have limited its widespread application [219]. Targeting pathogenic bacteria using bacteriophages or CRISPR-based microbiome editing represents an emerging strategy with potential benefits for livestock health and antibiotic reduction [220].

Despite these advancements, a direct, universally effective link between microbiota composition and host performance remains elusive. Future studies should focus on functional validation, longitudinal trials, and individualized microbiome interventions to bridge the gap between microbiome research and its real-world applications in livestock production.

## 8. Conclusions

Advancements in sequencing technologies have profoundly enhanced our understanding of the gut microbiota in livestock, shedding light on its pivotal role in digestion, nutrient metabolism, immune modulation, and overall health. This review has explored the microbiota of ruminants and monogastric livestock, summarizing the current state of the art while identifying critical gaps that merit further research.

One of the key takeaways is the need to expand microbiome studies beyond widely studied species and breeds, as the microbiota composition and its functional potential may differ significantly based on genetic background, geographical distribution, and farming systems. Future research should prioritize the exploration of microbial interactions within the gut ecosystem, particularly the interplay between bacterial communities, archaea, fungi, and phages, which remains underexplored. Understanding these dynamics is essential for leveraging microbiota-driven strategies to enhance feed efficiency, improve livestock productivity, and mitigate environmental impact.

The growing field of microbiota manipulation through dietary interventions, probiotics, and microbial transplantation presents promising avenues for improving animal health and reducing reliance on antibiotics in livestock production. Despite significant progress, challenges persist in standardizing methodologies for microbiome studies, particularly in sampling procedures and bioinformatics pipelines. Addressing these challenges will enable more reproducible and comparable results across studies, ultimately leading to a more comprehensive understanding of how gut microbiota can be harnessed for sustainable livestock production. In this context, interdisciplinary collaboration between microbiologists, veterinarians, nutritionists, and data scientists will be crucial for advancing microbiome research and translating findings into practical applications for the livestock industry.

## Data Availability

No new data were created or analysed in this study.
